# Loss of S100A1 expression leads to Ca^2+^ release potentiation in mutant mice with disrupted CaM and S100A1 binding to CaMBD2 of RyR1

**DOI:** 10.14814/phy2.13822

**Published:** 2018-08-12

**Authors:** Erick O. Hernández‐Ochoa, Zephan Melville, Camilo Vanegas, Kristen M. Varney, Paul T. Wilder, Werner Melzer, David J. Weber, Martin F. Schneider

**Affiliations:** ^1^ Department of Biochemistry and Molecular Biology University of Maryland School of Medicine Baltimore Maryland; ^2^ Center for Biomolecular Therapeutics (CBT) University of Maryland School of Medicine Maryland; ^3^ Institute of Applied Physiology Ulm University Ulm Germany

**Keywords:** Ca^2+^ release, calmodulin, excitation‐contraction coupling, isothermal calorimetry, *RyR1*, S100A1, skeletal muscle

## Abstract

Calmodulin (CaM) and S100A1 fine‐tune skeletal muscle Ca^2+^ release via opposite modulation of the ryanodine receptor type 1 (RyR1). Binding to and modulation of RyR1 by CaM and S100A1 occurs predominantly at the region ranging from amino acid residue 3614‐3640 of RyR1 (here referred to as CaMBD2). Using synthetic peptides, it has been shown that CaM binds to two additional regions within the RyR1, specifically residues 1975‐1999 and 4295‐4325 (CaMBD1 and CaMBD3, respectively). Because S100A1 typically binds to similar motifs as CaM, we hypothesized that S100A1 could also bind to CaMBD1 and CaMBD3. Our goals were: (1) to establish whether S100A1 binds to synthetic peptides containing CaMBD1 and CaMBD3 using isothermal calorimetry (ITC), and (2) to identify whether S100A1 and CaM modulate RyR1 Ca^2+^ release activation via sites other than CaMBD2 in RyR1 in its native cellular context. We developed the mouse model (RyR1D‐S100A1KO), which expresses point mutation RyR1‐L3625D (RyR1D) that disrupts the modulation of RyR1 by CaM and S100A1 at CaMBD2 and also lacks S100A1 (S100A1KO). ITC assays revealed that S100A1 binds with different affinities to CaMBD1 and CaMBD3. Using high‐speed Ca^2+^ imaging and a model for Ca^2+^ binding and transport, we show that the RyR1D‐S100A1KO muscle fibers exhibit a modest but significant increase in myoplasmic Ca^2+^ transients and enhanced Ca^2+^ release flux following field stimulation when compared to fibers from RyR1D mice, which were used as controls to eliminate any effect of binding at CaMBD2, but with preserved S100A1 expression. Our results suggest that S100A1, similar to CaM, binds to CaMBD1 and CaMBD3 within the RyR1, but that CaMBD2 appears to be the primary site of RyR1 regulation by CaM and S100A1.

## Introduction

In excitation‐contraction coupling (ECC) of muscle, depolarization of the sarcolemma (normally induced by action potentials) causes Ca^2+^ to be released from the sarcoplasmic reticulum (SR) (Sandow 1952; Numa et al. 1990). This in turn triggers muscle contraction. The sarco/endoplasmic reticulum Ca^2+^ ATPase (SERCA) actively sequesters calcium from the cytoplasm back into the SR (Ebashi 1976). The depolarizing event alters the conformational state of the voltage‐dependent L‐type Ca^2+^ channels (Cav1.1) within the t‐tubular membrane (Schneider and Chandler 1973; Rios and Brum 1987), which in skeletal muscle directly activates the Ca^2+^ release channel (RyR1), a homotetrameric protein of 2.2 MDa within the SR membrane (Meissner et al. 1986; Imagawa et al. 1987; Block et al. 1988). This tight regulation of Ca^2+^ release is modulated by numerous intracellular ligands (Meissner 2002; Lanner et al. 2010) that interact with the large cytoplasmic region of RyR1 (Radermacher et al. 1992; Zalk et al. 2015). The skeletal muscle RyR1 is inhibited by calmodulin, CaM (Chen and Maclennan 1994; Fuentes et al. 1994; Buratti et al. 1995; Tripathy et al. 1995; Moore et al. 1999; Balshaw et al. 2002; Yamaguchi et al. 2011) and potentiated by S100A1 (Baudier and Gerard 1983; Kato and Kimura 1985; Haimoto and Kato 1987; Fano et al. 1989; Maco et al. 1997; Treves et al. 1997; Prosser et al. 2008).

CaM is a 17 kDa cytosolic Ca^2+^‐binding protein that regulates multiple calcium‐dependent processes including ion channel function (Balshaw et al. 2002; Bers 2011; Ben‐Johny and Yue 2014). CaM contains four EF‐hand Ca^2+^‐binding pockets (two in the amino‐terminal domain and two in the carboxy‐terminal domain) (Babu et al. 1985; Chattopadhyaya et al. 1992). In skeletal muscle activation, CaM weakly activates RyR1 at resting myoplasmic Ca^2+^ levels and inhibits RyR1 activation at high calcium levels (Rodney 2008; Bers 2011). Binding assays, site‐directed mutagenesis, and cryo‐EM studies have shown that RyR1 has a single high‐affinity CaM binding site per RyR1 monomer (4 CaM/RyR1) at amino acid residues 3614‐3640; here referred to as CaMBD2 (Takeshima et al. 1989; Moore et al. 1999; Balshaw et al. 2001; Samso and Wagenknecht 2002; Yamaguchi et al. 2003, 2004; Zhu et al. 2004).

S100A1, a 21 kDa dimeric molecule is characterized by its EF‐hand Ca^2+^‐binding motifs (Moore 1965; Baudier and Gerard 1983; Baudier et al. 1983; Isobe et al. 1983), each hand displaying a helix‐loop‐helix arrangement (Baudier and Gerard 1983; Baldisseri et al. 1999; Rustandi et al. 2002; Melville et al. 2017a). S100A1 expression is tissue‐specific and found mostly in heart, skeletal muscle, and brain with decreasing abundance in this order (Kato and Kimura 1985). Its expression in excitable cells provides a unique environment to execute and fine tune important calcium‐related processes (Schaub and Heizmann 2008). Its role has been well defined in cardiac muscle as an enhancer of Ca^2+^ release and contractility as well as a possible therapeutic agent (Most et al. 2001, 2003a, 2005; Schaub and Heizmann 2008; Wright et al. 2009). The elimination of S100A1 in cardiac cells resulted in suppression of global myoplasmic Ca^2+^ transients and depressed activation of sarcoplasmic reticulum Ca^2+^ release following *β*‐adrenergic stimulation and sarcolemmal Ca^2+^ influx (Du et al. 2002; Most et al. 2006). In skeletal muscle, S100A1 regulates Ca^2+^ release and muscle contraction via multiple mechanisms including RyR1 modulation (Adhikari and Wang 2001; Most et al. 2003b; Prosser et al. 2008, 2010; Heiny 2010; Volkers et al. 2010).

Similar to CaM, various studies indicate that S100A1 regulates skeletal muscle RyR1 by binding to CaMBD2 (Prosser et al. 2008, 2011; Wright et al. 2008; Heiny 2010; Bers 2011; Yamaguchi et al. 2011). A single RyR1 amino acid substitution L3625D in the CaMBD2 results in the loss of S100A1 potentiation and CaM inhibition (Yamaguchi et al. 2011). Binding of CaM and S100A1 to CaMBD2 is conserved between skeletal and cardiac RyR isoforms (Yamaguchi et al. 2003, 2004, 2011; Lau et al. 2014). CaMBD2 has been described as the primary CaM/S100A1‐binding domain and it has been the subject of intense research (Prosser et al. 2011; Yamaguchi et al. 2011; Lau et al. 2014). However, several studies suggest that CaM (and other Ca^2+^‐binding proteins, including S100A1) could bind to and regulate RyR1 at different CaMBDs. Indeed, studies using fusion proteins and synthetic peptides have unveiled additional CaM‐binding sites within RyR1: CaMBD1, residues 1975‐1999 and CaMBD3, residues 4295‐4325 (Chen and Maclennan 1994; Guerrini et al. 1995; Lau et al. 2014). While many studies have identified CaM/S100A1‐binding to the RyR1 region CaMBD2 and corresponding modulation in vitro, in vivo studies related to newly identified CaMBD1 and CaMBD3 and their role in ECC are limited.

**Figure 1:**
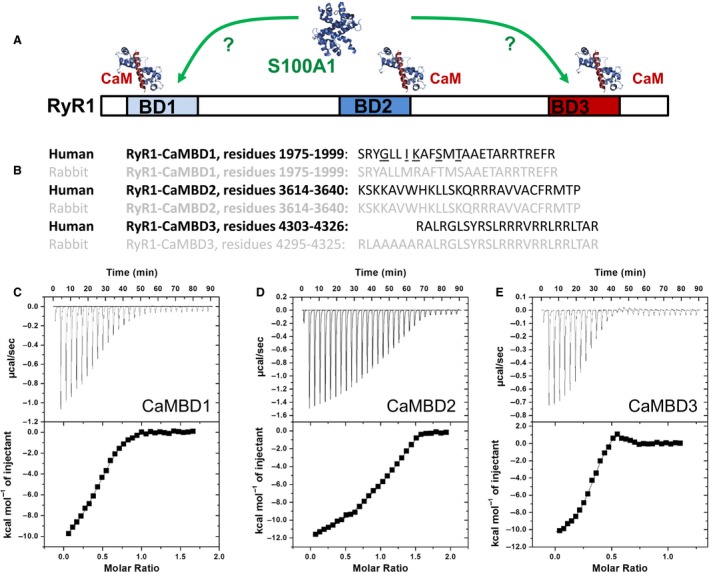
Isothermal titration calorimetry of the interaction of Ca^2+^/S100A1 with CaMBD1‐3 peptides. (A) Cartoon representation of CaMBD sites 1‐3 on RyR1. (B) peptides of CaMBD1‐3 from human RyR1 compared with rabbit RyR1s sequences. (C–D) ITC titration heat plots show interaction between S100A1 and CaMBDs in the presence of 10 mmol/L CaCl_2_. Symbols are heat integrals for each peak plotted vs. molar ratio. Solid lines through the symbols are best fits using a two binding site model (see Table 1 for derived thermodynamic parameters).

**Figure 2:**
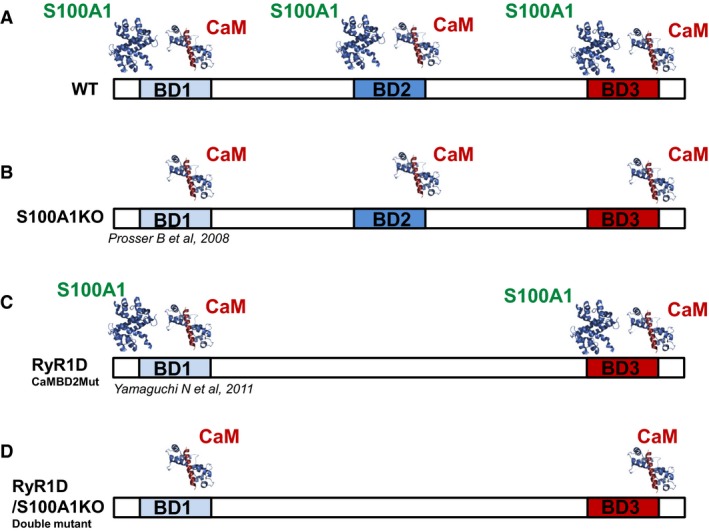
Schematic representation of CaMBD sites 1‐3 on RyR1 and their ligand interaction. (A) wild‐type (WT): CaM and S100A1 can bind to CaMBD1‐3 located in the RyR1. (B) S100A1KO: CaM can bind to CaMBD1‐3 in the absence of S100A1. (C), RyR1D: neither CaM nor S100A1 bind to CaMBD2, S100A1 can compete with CaM for the other two sites. (D) RyR1D‐S100A1KO: Double mutant that lacks the CaMBD2 site and the expression of S100A1, CaM can bind to the other two sites.

There is growing evidence that S100A1 plays a crucial role in fine tuning skeletal muscle Ca^2+^ release along with the more ubiquitous modulator CaM, suggesting a unique relationship between these two calcium binding proteins which is yet to be fully defined (Bers 2011). As S100A1 frequently binds to similar structural motifs as CaM (Baudier et al. 1987; Rhoads and Friedberg 1997), it is possible that S100A1 also binds to CaMBD1 and CaMBD3. The aim of our study was twofold: (1) to establish whether S100A1 binds to CaMBD1 and CaMBD3 using isothermal calorimetry (Fig. 1A) and (2) to identify whether S100A1 and CaM modulate Ca^2+^ release activation via sites other than CaMBD2 in full length RyR1 in its native cellular context, by monitoring action potential‐induced Ca^2+^ signals in single muscle fibers from the RyR1D‐S100A1KO (global S100A1 knock‐out mice) mouse model (see Fig. 2D). We crossbred RyR1D (Yamaguchi et al. 2011) and S100A1KO (Prosser et al. 2008) mice to obtain homozygous double‐mutant RyR1D‐S100A1KO mice. In this model, RyR1 cannot be regulated via CaM or S100A1 at CaMBD2 and it will be deficient of S100A1, allowing only CaM to bind to other putative‐binding sites within the RyR1. Observations made in this double‐mutant mice, RyR1D‐S100A1KO, are compared with those in the RyR1D‐mutant mice by testing the biophysical characteristics of Ca^2+^ release using in‐cellulo assays and in vitro isothermal calorimetry to test for binding of S100A1 to CaMBD sites.

## Materials and Methods

### Generation of transgenic mice

The RyR1D mouse (129/SvEv genetic background) containing the L3625D mutation, that impairs CaMBD2 function was described previously (Yamaguchi et al. 2011). The S100A1 knockout mouse (S100A1KO), maintained on a mixed C57BL/6–129S6 line has also been described (Prosser et al. 2008). RyR1D and S100A1KO were generous gifts from Drs. G. Meissner and D. Zimmer, respectively. Double mutant RyR1D‐S100A1KO mice were obtained by crossbreeding homozygous RyR1D (Yamaguchi et al. 2011) and S100A1KO mice (Prosser et al. 2008). The two strains were interbred (to generation F12) to assure genetic homogeneity and to give rise to the four double homozygous strains used in our experiments: (1) wild‐type (WT)/WT, (2) WT/S100A1KO, (3) RyR1D/WT and (4) RyR1D‐S100A1KO mice. Homozygous RyR1D, S100A1KO, and double‐mutant RyR1D‐S100A1KO mice are fully viable. All animals were kept at regular 12/12‐h light‐dark cycles and were given free access to water and food under pathogen‐free conditions. Animals were separated in cages depending on the age group and their sex. The mice were handled according to the National Institutes of Health guidelines for the use and care of experimental animals. All animal involving experiments were carried out in accordance to the Institutional Animal Care and Use Committee of the University of Maryland. RyR1D and S100A1KO genotypes were identified has previously described (Prosser et al. 2008; Yamaguchi et al. 2011).

### FDB fiber preparation

Six‐ to eight‐week‐old male mice were euthanized and the flexor digitorum brevis (FDB) muscles were dissected. Single myofibers from FDB muscles were enzymatically isolated in minimum essential medium (MEM) containing 1 *μ*L/mL Gentamicin (Sigma, St. Louis, MO; Cat. No. G1397) and 2 mg/mL type I collagenase (Sigma, Cat. No. C0130) for 3–5 h at 37°C as previously described (Liu et al. 1997). Solutions were filtered using a 0.2 *μ*m polyethersulfone membrane (http://www.thermoscientific.com, Cat. No. 90‐9920). Muscle fibers were then plated on glass‐bottomed dishes (Matek Cor. Ashland, MA, Cat. No. P35G‐1.0‐14‐C,) coated with laminin (Thermo Fisher, Rockford, IL, Cat. No. 23017‐015) and cultured (5% CO2; 37°C) for 12–18 h before experiments.

### Exclusion criteria

Before any experiments were carried out on dissociated FDB muscle fibers, we identified muscle fibers using specific criteria indicative of healthy functional cells. This includes morphological integrity (smooth surface, straight fiber, with clear striation pattern and no bents or waves), uniform responses to field stimulation and reproducibility of calcium transient responses (Hernandez‐Ochoa et al. 2016). Muscle fibers that did not respond or that did not respond equally to alternating polarities of electric field stimulation via two remote electrodes were discarded from the testing pool (Hernandez‐Ochoa et al. 2016).

### Rhod‐2 Ca^2+^ imaging

Rhod‐2 measurements were carried out on a high‐speed confocal system (LSM 5 Live system, Carl Zeiss, Jena, Germany) as previously described (Hernandez‐Ochoa et al. 2016). Myofibers were loaded with 1 *μ*mol/L rhod‐2 AM (Thermo Fisher, Cat. No. R‐1245MP) in L‐15 media (Life Technologies, Carlsbad, CA, Cat. No. 21083027). The ionic composition of L‐15 in mmol/L is: 137 NaCl, 5.7 KCl, 1.26 CaCl2, and 1.8 MgCl2, pH 7.4). The L‐15 medium was supplemented with 0.25% w/v bovine serum albumin (BSA; Sigma‐Aldrich, St Louis MO, Cat. No. A‐7906) and applied for one hour at room temperature to allow dye loading. The fibers were then washed thoroughly to remove residual Rhod‐2 AM and incubated with plain L‐15, warmed to room temperature (20–22°C) for 20 min to allow dye conversion. At this point the culture dish was mounted on the Zeiss Axiovert 200M inverted microscope of the Zeiss LSM 5 Live confocal system. Imaging was performed with a 60 × /1.3 NA water‐immersion objective lens. Excitation for rhod‐2 was provided by the 532‐nm line of a 100‐mW diode laser, and emitted light was collected at >550 nm. Supramaximal field stimulation (1 msec square pulse, 30 V/cm) was produced by a custom pulse generator and applied via two platinum wires positioned perpendicular to the bottom of the dish, ∼5 mm apart, to elicit action potentials. Muscle fibers were centrally positioned relative to the electrodes and to the field of view, at an angle of less than about ±45° relative to an imaginary line between the tips of the electrodes. Only fibers exhibiting reproducible all or none responses to field stimulation of alternating polarity were used for the analysis. The field stimulus was applied 100 msec after the start of the confocal scan sequence, thus providing control images before stimulation at the start of each recording sequence. These control images were used to determine the resting steady‐state fluorescence level (F0). Average intensity of fluorescence within selected regions of interest (ROIs) within a myofiber was measured with Zeiss LSM Image Examiner (Carl Zeiss, Jena, Germany). Images in line scan (*x*‐*t*) mode (frame size: 512 pixels in *x* × 10,000 pixels in *t*; scan speed: 100 *μ*sec/line for 1 sec duration of acquisition were background corrected by subtracting an average value recorded outside the cell. The average F0 value in each ROI before electrical stimulation was used to scale Ca^2+^ signals in the same ROI as ΔF/F0. Here, we used rhod‐2, a high‐affinity dye. Based on calibration data, rhod‐2 was at most 40% saturated with Ca^2+^. The length of the fibers used was 400–600 *μ*m, and the width was 25–60 *μ*m. Ca^2+^ imaging experiments were carried out at room temperature.

### Indo‐1 Ca^2+^ imaging

RyR1D and RyR1D‐S100A1KO muscle fibers were loaded with indo‐1 using same loading procedure described for rhod‐2 and processed as previously described (Robison et al. 2017). Indo‐1 resting signals were imaged on an Olympus IX71 inverted microscope (Olympus, Center Valley, PA) with an Olympus 60× water immersion objective. Indo‐1 fluorescence emission was detected at 405 and 485 nm simultaneously, using two parallel photomultipliers with a sampling rate of 2 kHz. Excitation light from a broadband UV source was filtered through a 380 ± 10 nm band pass filter, and emission was split using a dichroic mirror and passed through either a 405 ± 10 or 485 ± 10 nm bandpass filter.

### Calculation of SR Ca^2+^ release flux from rhod2‐AM fluorescence recordings

A Ca^2+^ removal model including binding and transport was used to estimate the time course of the Ca^2+^ release flux during action potential induced activation as described (Melzer et al. 1987; Baylor and Hollingworth 2003). Binding to Ca^2+^‐specific sites of troponin C (T‐sites) and parvalbumin‐like Ca^2+^‐Mg^2+^ sites (P‐sites) was calculated, using binding site concentrations and rate constants of troponin and parvalbumin adopted from (Baylor and Hollingworth 2003). The rate constants were adjusted to our experimental temperature as previously described (Prosser et al. 2010; Yamaguchi et al. 2011). Fast Ca^2+^ binding to ATP was described by a component proportional to free Ca^2+^ times a scaling factor of 3.6 (Prosser et al. 2010). Ca^2+^ removal by transport (e.g., uptake by the SR Ca^2+^‐ATPase) was assumed to be proportional to free [Ca^2+^] (rate constant k_uptake_ 1000/sec). The fixed rate constant values used for the calculations were for T‐sites: kon, T, Ca = 115 *μ*M/sec, koff, T, Ca = 150/sec; and for P‐sites: kon,P,Ca = 54.0 *μ*M/sec, koff, P, Ca = 0.65/sec, kon, P, Mg = 0.043 *μ*M/sec, koff, P, Mg = 3.9/sec, kuptake = 1000/sec. [T]tot and [P]tot, the total concentrations of T‐sites and P‐sites, were 0.240 mmol/L and 1.5 mmol/L, respectively. After an initial calculation with these parameters, kon, P, Mg, and kuptake were adjusted by iteration to minimize the least squares deviation between calculated and measured fluorescence ratio. The Ca^2+^ occupancies of all model compartments [T‐sites, P‐sites, ATP (F‐sites), and uptake] were summed, and the release flux was calculated as the time derivative of the sum (Timmer et al. 1998). Calculations were performed using Euler's method (Scarborough 1966). Analysis was performed using Excel Solver (Microsoft).

### Peptides

All peptides were synthesized using solid‐state peptide synthesis and their purity was determined to be >95% by high pressure liquid chromatography and mass spectrometry (Biosynthesis Inc., Lewisville, TX or GenScript, Piscataway, NJ). Three peptides derived from human RyR1 sequence were synthesized: CaMBD2, residues 3614‐3640, KSKKAVWHKLLSKQRRRAVVACFRMTP; CaMBD1, residues 1975‐1999, SRYGLLIKAFSMTAAETARRTREFR; and CaMBD3, residues 4303‐4326, RALRGLSYRSLRRRVRRLRRLTAR. As they are internal peptides the C‐terminus was amidinated and N‐terminus was acetylated to neutralize the charged ends. The peptides stock were made in D_6_‐DMSO and their concentration were determined by quantitative amino acid analysis (Biosynthesis Inc., Lewisville, TX) and/or determined using the extinction coefficient for the methyl ester of *N*‐acetyl tryptophan, ε280 = 5600 cm^−1^M^−1^.

### Expression and purification of S100A1

Expression and purification (>95% by SDS‐PAGE) of recombinant human ^15^N‐labeled S100A1 was performed as previous described (Melville et al. 2017b). The concentrations of S100A1 stock solutions were determined using the Bio‐Rad Protein Assay (Bio‐Rad Inc., Hercules, CA) using a known concentration of BSA as the standard. Concentrations are given in S100A1 monomer concentration except where indicated.

### Isothermal titration calorimetry (ITC)

Heat changes during the titration of each CaMBD peptide into Ca^2+^‐S100A1 were measured using a VP‐ITC titration microcalorimeter (MicroCal, Inc., Northhampton, MA) as done previously (Wilder et al. 2003). All solutions were degassed under vacuum and equilibrated at 37°C prior to titration. For each titration the sample cell (1.4 mL) contained 10 mmol/L TES, pH 7.2, 15 mmol/L NaCl, and 10 mmol/L CaCl_2_, 0.5 mmol/L TCEP, <5% D_6_‐DMSO (from peptide stocks or equivalent D_6_‐DMSO added to controls), and 0.04 mmol/L CaMBD peptide, while the reference cell contained water. Upon equilibration, 0.20–0.35 mmol/L S100A1 (dimer concentration) prepared in the same buffer without peptide but equivalent D_6_‐DMSO was injected using the default injection rate with a time interval between each injection to allow the sample to return to baseline. The resulting titration curves were corrected for protein‐free buffer control.

### Data analysis

Images were collected and evaluated using the same settings and enhancing parameters so that all images could be directly compared. Line‐scan images were analyzed using LSM examiner (Carl Zeiss, Jena, DE). Calculation of AP‐induced Ca^2+^ signals were conducted using Origin Pro 8 (OriginLab Corporation, Northampton, MA, USA) and SPSS for Windows ver. 24.0 (SPSS Inc., Chicago, IL). Summary data were reported as mean ± SD. Normal distribution of data was assessed using the Kolmogorov–Smirnov test. Unpaired two sample Student's *t*‐test was used to test for differences between the means of the indo 1‐resting values and peak AP‐induced Ca^2+^ transients from two different samples. ITC results were background buffer subtracted and analyzed using the Origin for ITC software (MicroCal, Northampton, MA) and fit using two‐site fitting model (Freire et al. 1990). Differences were considered significant when *P* < 0.05.

## Results

### Binding of S100A1 to CaMBDs1‐3

There is substantial evidence that CaM and S100A1 may bind to multiple sites within the RyR1. Using isothermal calorimetry Lau et al., demonstrated that CaM can bind to three different peptides from rabbit RyR1: rCaMBD1, residues 3614‐3640; rCaMBD2, residues 1975‐1999; rCaMBD3, residues 4295‐4325, see Fig. 1A) (Lau et al. 2014). Interestingly, Treves et al. described three potential S100A1‐binding sites on RyR1 (Treves et al. 1997). The S100A1 site 1 of (Treves et al. (1997) encompasses the CaMBD1 described by Lau et al. (2014) and evaluated here; while their S100A1 sites 2 and 3 do not correspond, but are near to CaMBD 2 and 3 studied here.

S100 proteins often bind to similar structural regions as CaM (Baudier et al. 1987; Rhoads and Friedberg 1997), therefore, we used isothermal titration calorimetry (ITC) to analyze the binding of human S100A1 to peptides from the 3 CaMBDs in human RyR1: CaMBD2, residues 3614‐3640; CaMBD1, residues 1975‐1999; CaMB3, residues 4303‐4326 (Fig. 1B). In the presence of 10 mmol/L Ca^2+^, to assure all four Ca^2+^‐binding sites were occupied, S100A1 bound to all three RyR1 peptides. Since S100A1 is a homodimer, it has two identical binding sites that are exposed upon Ca^2+^‐binding and typically short peptides will bind identically to each site. However, in this case the ITC data shows two peptide sites per S100A1 dimer with differing affinities (Fig. 1C–E; see Table 1). CaMBD3 exhibited the tightest S100A1 binding (K_D_ of ~9 nmol/L & ~7 *μ*mol/L), CaMBD2 was next (K_D_ of ~146 nmol/L & ~17 *μ*mol/L), and CaMBD1 showed the weakest affinity (K_D_ of ~1000 nmol/L and ~21 *μ*mol/L). The difference in affinity between the two‐sites on S100A1 was greater for the peptides with the higher affinities: CaMBD3, CaMBD2, and CaMBD1 showed 755‐fold, 114‐fold, and 16‐fold difference between the tight and weak peptide‐binding site, respectively. It is possible that S100A1 binds in a different way to a full‐length RyR1 protein target, than to short peptides as used here. Only one of the S100A1 sites may be occupied, or, if both sites participate, they may bind to different targets simultaneous rather than to a single one.

**Table 1:** Results of isothermal titration calorimetry of S100A1 association with RyR1 CaMBD peptides

Peptide (site)	N (sites)	ΔH (kcal/mol)	−TΔS (kcal/mol)	ΔG (kcal/mol)	K_D_ (nM)
CaMBD1 (1)	1.14 ± 0.10	−4.5 ± 0.7	1.89 ± 0.26	−8.4 ± 0.1	1303.4 ± 284.0
CaMBD1 (2)	0.75 ± 0.07	−10.8 ± 0.4	0.62 ± 0.03	−6.7 ± 0.2	2.1 × 10^4^ ± 6881
CaMBD2 (1)	0.78 ± 0.09	−11.4 ± 0.2	0.85 ± 0.00	−9.7 ± 0.1	146.3 ± 23.9
CaMBD2 (2)	0.51 ± 0.15	−10.3 ± 3.0	0.70 ± 0.23	−6.8 ± 0.1	1.7 × 10^4^ ± 2635
CaMBD3 (1)	1.79 ± 0.08	−3.6 ± 0.7	3.40 ± 0.51	−11.9 ± 1.2	8.9 ± 11.0
CaMBD3 (2)	1.27 ± 0.07	−6.1 ± 0.9	1.22 ± 0.16	−7.4 ± 0.2	6.7 × 10^3^ ± 2166

Each titration was run in triplicate (*n* = 3) so the value shown is the average ± standard deviation. H = enthalpy; S = entropy; T = absolute temperature (Kelvin); G = Gibbs free energy; K_D_ = dissociation constant).

### RyR1D‐S100A1KO mouse model

Previous results have shown that S100A1 and CaM can regulate RyR1 activity via binding to CaMBD2 (Prosser et al. 2008; Wright et al. 2008; Yamaguchi et al. 2011). The preceding section (Fig. 1 C–E) allows us to conclude that S100A1 can bind to CaMBD1‐3 peptides from rabbit RyR1. An important question is whether S100A1 and/or CaM modulates Ca^2+^ release activation via sites other than CaMBD2 in full length RyR1 in its native cellular context. Our approach to test this hypothesis was to eliminate the regulation by CaM and S100A1 at CaMBD2 via L3625D mutation in CaMBD2 (Yamaguchi et al. 2011), and test the role of eliminating S100A1 in this context (Prosser et al. 2008). We tested the effect of eliminating the expression of S100A1 in skeletal muscle fibers with L3625D mutation in CaMBD2 on Ca^2+^ homeostasis and action potential‐induced Ca^2+^ release. To test this hypothesis, we developed the RyR1D‐S100A1KO mouse model. This mouse contains a mutation in CaMBD2 that disrupts strong RyR1 modulation by CaM and S100A1 (RyR1D mice, Fig. 2C, (Yamaguchi et al. 2011)) and is deficient of S100A1 (Fig. 2B, (Prosser et al. 2008)). In this scenario, Ca^2+^ release channels from RyR1D‐S100A1KO fibers (Fig. 2D) might be regulated by CaM at either CaMBD1 and/or CaMBD3 (Fig. 2D), but not by S100A1 at any site. In contrast, the single transgenic RyR1D mice cannot be modulated by either CaM or S100A1 at CaMBD2, but can be modulated by both CaM and S100A1 at CaMBD1 and/or 3. Muscle fibers from the individual homozygous mutant genotypes (i.e., RyR1D or S100A1KO) displayed the characteristic features described earlier (i.e., reduction in the amplitude of Ca^2+^ transients, Ca^2+^ treppe in RyR1D muscle fibers when compared to WT counterparts (Bers 2011; Prosser et al. 2011; Heiny 2010)). Double‐mutant genotypes progressed with normal lifespan compared to S100A1KO, RyR1D, and WT mice. There were no obvious changes in phenotype including size, body weight, and behavior.

### Resting myoplasmic Ca^2+^ in RyR1D‐S100A1KO muscle fibers is unaltered

Because S100A1 can bind to CaMBDs 1 and 3, and S100A1 has been shown to regulate Ca^2+^ signaling in striated muscle (Most et al. 2003b; Prosser et al. 2008, 2011) we next investigated the calcium homeostasis in single muscle fibers from RyR1D‐S100A1KO and RyR1D mice using the ratiometric Ca^2+^ indicator indo‐1. We found that RyR1D‐S100A1KO and RyR1D muscle fibers had resting indo‐1 ratios that were not significantly different (RyR1D, 0.60 ± 0.06, *n* = 24 fibers vs. RyR1D‐S100A1KO, 0.61 ± 0.07, *n* = 20; *P* = 0.76, two sample *t* test), indicating that resting calcium concentration was similar in both types of fibers.

### Action potential‐induced Ca^2+^ transients are increased in RyR1D‐S100A1KO compared to RyR1D muscle fibers

**Figure 3:**
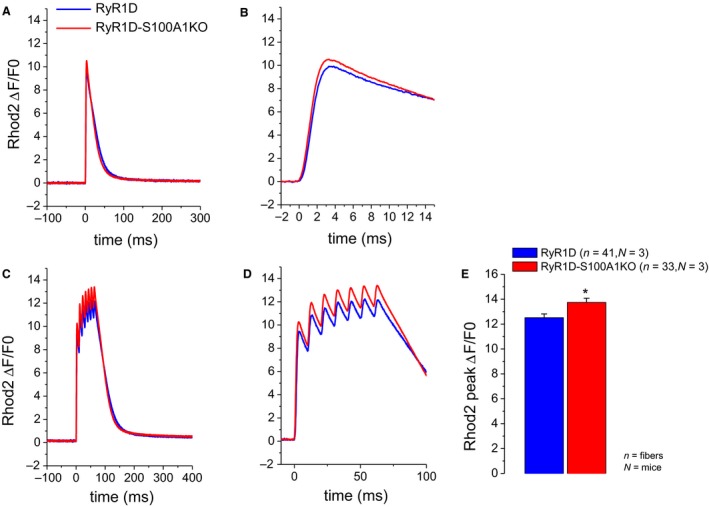
Action potential‐induced Ca^2+^ transients in RyR1D and RyR1D/S100A1KO fibers. (A) average change in rhod‐2 fluorescence in RyR1D (*n* = 41, *N* = 3) and RyR1D‐S100A1KO (*n* = 33, *N* = 3) fibers in response to a single action potential elicited by field stimulation. (B) Expanded view of panel (A) showing minor differences in peak fluorescence between RyR1D and RyR1D‐S100A1KO fibers. (C) Average change in rhod‐2 fluorescence in RyR1D and RyR1D‐S100A1KO fibers in response to repetitive stimulation [100 Hz, 66 msec]. (D) expanded view of panel (C). (E) Mean maximal change in rhod2 fluorescence at tetanic stimulation for RyR1D and RyR1D‐S100A1KO fibers. Using repetitive stimulation, a modest but significant increase in the amplitude of the Ca^2+^ transients was found in RyR1D‐S100A1KO fibers compared to RyR1D controls, ΔF/F0: RyR1D = 12.51, versus RyR1D‐S100A1KO = 13.74; *Two sample *t* test, *P *=* *0.0078.

To determine if RyR1D‐S100A1KO muscle fibers exhibited alterations in action potential‐induced Ca^2+^ transients, a critical step of the ECC mechanism, we next monitored rhod‐2 fluorescence transients elicited by field stimulation. We measured responses following repetitive electrical stimulation of RyR1D‐S100A1KO and RyR1D muscle fibers using an ultra‐high‐speed line‐scanning confocal microscope (100 *μ*sec/line). Figure 3 shows average responses from 41 RyR1D and 33 RyR1D‐S100A1KO double mutant fibers stimulated by a single action potential (Fig. 3A) or by a train of 5 action potentials at 50 Hz (Fig. 3C). Counterintuitively, we found larger calcium transients in RyR1D‐S100A1KO which lack S100A1, the known calcium enhancer at CaMBD2, compared to RyR1D FDB fibers, which express S100A1. Single electrically induced calcium transients (Fig. 3A–B) showed no difference in amplitude between RyR1D‐S100A1KO (red trace) and RyR1D (blue trace). In Figure 3D we expanded the time scale to show a trend of increasing transients in RyR1D‐S100A1KO fibers during repetitive stimulation. This increase closely resembles a physiologically relevant pattern seen during contractile muscle activation. Here the average peak of rhod‐2 fluorescence at the end of the stimulation shows a modest, but significant, increase in RyR1D‐S100A1KO (*n* = 33) compared to RyR1D (*n* = 41) (*P* = 0.0078, two sample *t* test). Figure 3E shows a statistically significant increase in amplitude of rhod‐2 peak for the last pulse of the train.

### AP‐induced Ca^2+^ release flux is enhanced in RyR1D‐S100A1KO fibers

**Figure 4:**
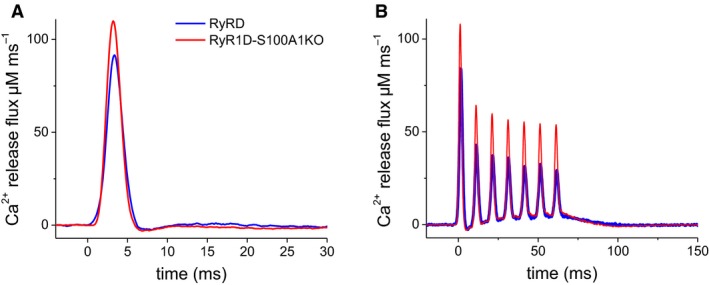
RyR1D‐S100A1KO fibers exhibit enhanced SR Ca^2+^ release. Average SR Ca^2+^ release flux of RyR1D and RyR1D‐S100A1KO fibers, estimated using a Ca^2+^ removal model. Ca^2+^ release flux trajectories were calculated from rhod2 transients shown in Figure 3A and C, elicited by a single (A) or a train of action potentials (B) demonstrating enhancement in RyR1D‐S100A1KO fibers compared to RyR1D controls. SR, sarcoplasmic reticulum.

To evaluate the alterations in SR Ca^2+^ release corresponding to the increase in rhod‐2 fluorescent Ca^2+^ transients during repetitive action potential firing in RyR1D‐S100A1KO‐mutant fibers, we fitted a Ca^2+^ removal model to the measurements (Melzer et al. 1987; Baylor and Hollingworth 2003; Prosser et al. 2010). The rate of SR Ca^2+^ release was calculated as the time derivative of free, bound, and pumped Ca^2+^obtained from the model fit. The average SR Ca^2+^ release flux time courses for RyR1D‐S100A1KO and RyR1D fibers are presented in Figure 4. Peak initial flux from a single AP was increased by 19% in RyR1D‐S100A1KO fibers compared with RyR1D counterparts (Fig. 4A). The Ca^2+^ release flux was consistently enhanced throughout the repetitive stimulation in RyR1D‐S100A1KO mutant fibers compared with RyR1D fibers (Fig. 4B). Both genotypes showed partial inactivation of release flux that approached a steady state during the trains of stimuli. The flux amplitude was suppressed by about 50% at the end of the 50 Hz train relative to the first pulse during the train in RyR1D‐S100A1KO fibers compared with 64% in RyR1D fibers, suggesting that RyR1D‐S100A1KO display less cumulative inactivation (Fig. 4B).

## Discussion

CaM and S100A1 are expressed in multiple organisms and tissues including striated muscle, where they are important Ca^2+^‐dependent modulators of numerous physiological functions (Schaub and Heizmann 2008). CaM and S100A1 bind to and modulate the activity of voltage‐gated ion channels and intracellular Ca^2+^ release channels (Balshaw et al. 2002; Ben‐Johny et al. 2015) and affect ECC in both skeletal and cardiac muscle (Most et al. 2001; Remppis et al. 2002; Wright et al. 2009; Prosser et al. 2011). The mechanism of CaM‐ and S100A1‐dependent regulation of RyR1 Ca^2+^ release channels is not clear. Numerous biochemical studies using RyR1 peptides have shown that CaM binds to multiple regions within RyR1 (Chen and Maclennan 1994; Menegazzi et al. 1994). Recently, a more quantitative structural biology approach validated three of these binding sites, CaMBD1‐CaMBD3 (Lau et al. 2014). S100 proteins, including S100A1 typically interact with CaM‐binding domains (Baudier et al. 1987; Rhoads and Friedberg 1997). A multidisciplinary approach, using transgenic mice, in vitro and in cellulo assays, structural biology and whole animal muscle physiology, has shown that S100A1 binds to CaMBD2 and regulates RyR1 (Prosser et al. 2008; Wright et al. 2008; Yamaguchi et al. 2011). However, interaction of S100A1 with CaMBD1 and CaMBD3 has *not* yet been evaluated.

Here, using ITC, we show that S100A1 can bind to all three CaM‐binding domains in RyR1 (CaMBD1‐3). ITC data from Lau et al. (2014), suggest that the affinity of CaM for these sites is CaMBD2 > CaMBD3 > CaMBD1. Therefore, it is likely that in RyR1D‐S100A1KO fibers, CaM binds to CaMBD1 and/or ‐3 resulting in RyR1 increased activity. Our ITC results show that S100A1 can also bind to all three CaMBDs, although with lower affinities compared to CaM. Similar to CaM, the affinity sequence was CaMBD2 > CaMBD3 > CaMBD1. Given the affinity of S100A1 for the CaMBDs, it is likely that the observed RyR1 reduced activity in RyR1D fibers, results from S100A1 binding to CaMBD3 and/or CaMBD1.

Our studies using muscle fibers from the double‐mutant RyR1D‐S100A1KO mice, which do not express S100A1 and lack RyR1 regulation by CaM or S100A1 binding at CaMBD2, showed no difference in resting myoplasmic Ca^2+^, indicating negligible effects on Ca^2+^ homeostasis at rest. However, we observed a modest but significant enhancement of the action‐potential induced Ca^2+^ transients and underlying Ca^2+^ release when compared to RyR1D fibers, which lack regulation by S100A1 or by CaM at CaMBD2 but do express S100A1. The potentiation of Ca^2+^ release seen in RyR1D‐S100A1KO fibers compared to RyR1D fibers may arise from CaM binding to CaMBD1 and/or CaMBD3.

Binding assays suggest that at elevated intracellular Ca^2+^ concentrations (100 *μ*mol/L) CaM binds to RyR subunits with a 1:1 stoichiometry (4 CaM per channel complex; (Tripathy et al. 1995; Yang et al. 1994)). This raises the question why there are three sites per subunit that can all bind CaM and S100A1? Our previously published observations regarding CaMBD2 (Prosser et al. 2008; Wright et al. 2008; Yamaguchi et al. 2011) and the present functional and ITC data suggest that CaMBD2 is the predominant binding site for CaM and S100A1. However, previous CaM‐binding assays also showed that the tetrameric channel complex can bind 8‐16 CaM molecules with nanomolar affinity in the presence of Ca^2+^ at concentration close to resting values (100 nM) (Yang et al. 1994; Tripathy et al. 1995). We hypothesize that at rest, CaMBD1 and −3 work as “Ca^2+^‐binding protein sponges,” allowing local enrichment of CaM and/or S100A1. During muscle activity and the corresponding increase in intracellular Ca^2+^ concentration, predominantly CaMBD2 is exposed, and final occupancy of this site depends on the respective local availability of CaM and S100A1.

Recent in vitro FRET (Rebbeck et al. 2016) and in silico studies (Scott and Kekenes‐Huskey 2016) suggested that high unphysiological (micromolar) concentration of S100A1 and extremely elevated intracellular Ca^2+^ levels (>100 *μ*mol/L) are required for RyR1 modulation by Ca^2+^/S100A1, respectively. In fast‐type skeletal muscle the intracellular concentration of S100A1 was reported to be ~3 *μ*mol/L (Haimoto and Kato 1987; Zimmer et al. 1991), with total intracellular concentration of CaM being about 5 *μ*mol/L (Robertson et al. 1981). If the intracellular levels of unbound S100A1 are comparable to those of CaM and if these proteins bind to identical target regions exhibiting similar affinities, then, a scenario of competition could explain some of our observations. At present we do not know the concentrations of CaM and S100A1 in the immediate vicinity of the RyR1. It seems possible that certain intracellular regions are enriched with either CaM or S100A1, as described for other intracellular signaling molecules such as cAMP (Bers and Ziolo 2001). The levels of CaM and S100A1 may also fluctuate based on muscle activity or disease conditions (Zimmer et al. 1997; Pertille et al. 2010). Furthermore highly localized intracellular Ca^2+^ microdomains, near the openings of Ca^2+^ channels (Llinas et al. 1992), may provide the elevated Ca^2+^ levels needed for S100A1 activation. These questions require further studies.

The observation that Ca^2+^ transient and Ca^2+^ release flux are increased when S100A1 is eliminated in RyR1D muscle fibers was contrary to what we expected since previous research demonstrated enhancement of Ca^2+^ release by S100A1 in many instances (Treves et al. 1997; Remppis et al. 2002; Most et al. 2003a; Prosser et al. 2008, 2010). Our results exemplify the versatility and specificity of these calcium‐binding proteins. During ECC in RyR1D‐S100A1KO muscle fibers, calmodulin or other related proteins might act as compensatory enhancers with higher affinity to CaMBD3 and CaMBD1 when CaMBD2 is not available. Our results suggest that like CaM, S100A1 binds to CaMBD1 and CaMBD3 to be able to exploit different systems for fine‐tuning the ECC pathway.

Finally, as suggested by Lau et al. (2014) another possible role for CaMBD1 and CaMBD3 could be in the regulation of the expression and trafficking of the RyR. This role has been assigned to CaM for voltage gated channels (Joiner et al. 2001). It would be interesting to develop mice with CaMBD1 and/or CaMBD3‐targeted mutations to further address the role of these accessory binding sites for CaM and S100A1 and their contribution to the function and trafficking of RyR1.

Overall, this study presents evidence for the existence of additional modulatory CaM/S100A1 sites (CaMBD1 and CaMBD3) in RyR1. Considering the tight regulation of Ca^2+^ within skeletal muscle, even the modest physiological effects observed for these CaMBDs could have important implications for the fine‐tuning of RyR1 under various activity‐dependent conditions.

## Conflict of Interest

No conflicts of interest, financial or otherwise, are declared by the authors.
